# Use of a Fundus Image-Based Titration Strategy for Selective Retina Therapy for Central Serous Chorioretinopathy

**DOI:** 10.3390/jcm13175230

**Published:** 2024-09-04

**Authors:** Seung Hee Jeon, Minhee Kim, Young-Jung Roh

**Affiliations:** 1 Department of Ophthalmology and Visual Science, Incheon St. Mary’s Hospital, Catholic University of Korea, 56, Dongsu-ro, Bupyeong-gu, Incheon 21431, Republic of Korea; jsh881107@hanmai.net; 2 Department of Ophthalmology and Visual Science, Yeouido St. Mary’s Hospital, Catholic University of Korea, 10, 63-ro, Yeongdeungpo-gu, Seoul 07345, Republic of Korea; chriszz@naver.com; 3 Threshold Co., Ltd., No. 1325, 40, 63-ro, Yeongdeungpo-gu, Seoul 07345, Republic of Korea

**Keywords:** central serous chorioretinopathy, fundus image-based treatment, macular sensitivity, selective retina therapy

## Abstract

**Background/Objectives:** This study evaluated the clinical outcomes of selective retina therapy (SRT) for treating central serous chorioretinopathy. A fundus image-based titration method was used for laser irradiation. **Methods:** This retrospective cohort study included 29 eyes (29 patients) that underwent SRT for CSC. Both the pulse energy and number of micropulses were adjusted according to the fundus image. Mean best-corrected visual acuity (BCVA), central foveal thickness (CFT), and subretinal fluid (SRF) height were measured 1, 2, 3, 4, and 6 months after SRT. Mean deviation (MD) was measured using microperimetry at 3 and 6 months post-treatment. **Results:** At 6 months after SRT treatment, SRF was completely resolved in 89.7% of cases (26/29 eyes). The mean Snellen BCVA significantly improved from 0.34 ± 0.31 logMAR (logarithm of the minimum angle of resolution) (20/40) at baseline to 0.24 ± 0.24 logMAR (20/32) at 6 months (*p* = 0.009). The 0.1 improvement in mean BCVA is equivalent to a 5-letter gain on the ETDRS chart. The mean CFT decreased significantly from 309.31 ± 81.6 μm at baseline to 211.07 ± 50.21 μm at 6 months (*p* < 0.001). The mean SRF height also decreased significantly from 138.36 ± 56.78 μm at baseline to 23.75 ± 61.19 μm at 6 months (*p* < 0.001). The mean MD was improved from −1.56 ± 1.47 dB at baseline to −1.03 ± 2.43 dB at 6 months (*p* = 0.07) after treatment. **Conclusions:** SRT using fundus image-based titration can yield favorable functional and anatomical outcomes in the treatment of CSC.

## 1. Introduction

Central serous chorioretinopathy (CSC) is a retinal disease characterized by idiopathic serous detachment of the neurosensory retina, which often occurs with or without pigment epithelial detachment (PED) [1]. The reported annual incidence of CSC is six times higher in men (9.9 per 100,000 individuals) than in women (1.7 per 100,000 individuals) [2]. Several risk factors including corticosteroids, stress, Type-A behavior, and genetic factors have been reported to be associated with the pathogenesis of CSC [3,4].

Although the pathophysiology of CSC is uncertain, the most likely mechanism involves increased choroidal permeability and elevated hydrostatic pressure, which trigger the development of PED and overpower the barrier function of the retinal pigment epithelium (RPE), resulting in the accumulation of subretinal fluid (SRF) [5,6]. This proposed mechanism is consistent with findings from previous imaging studies using fundus fluorescein angiography (FFA) and indocyanine green angiography (ICGA), which demonstrated extensive hyperdynamic, hyperpermeable choroidal circulation with localized areas of nonperfusion [7,8].

Acute CSC tends to be self-limiting and visual recovery is typically good within 1–4 months, even after a period of observation without any treatment [9]. Chronic or recurrent CSC results in reduced visual acuity and contrast sensitivity, or even permanent visual impairment in areas affected by SRF [10,11,12]. Therefore, SRF must be eliminated in patients with chronic CSC because the complete resolution of SRF promotes visual recovery and prevents further vision loss [13].

Although no standardized treatment exists for CSC, various modalities have been suggested, including conventional focal laser therapy targeting presumed RPE decompensation sites, photodynamic therapy (PDT), and intravitreal injection of vascular endothelial growth factor (VEGF) inhibitors [14]. While conventional laser treatment promotes the resolution of serous detachment, it carries the risk of central or paracentral scotoma and choroidal neovascularization (CNV) due to irreversible damage to the retina and choroid [15,16]. Although PDT has shown favorable results in many studies [17,18,19], adverse effects, such as acute vision loss due to RPE atrophy, have been reported following full- or reduced-dose PDT [20].

Selective retinal therapy (SRT) is a technique that was developed to prevent photoreceptor damage caused by conventional laser photocoagulation. SRT induces selective RPE damage without affecting the neural retina and promotes RPE rejuvenation by stimulating the migration and proliferation of RPE cells into irradiated areas, thereby enhancing metabolism in the treated region [21]. In SRT-treated areas, the release of cell mediators, such as matrix metalloproteinase-2 and pigment epithelium-derived factor, is known to facilitate RPE restoration [22,23].

In previous clinical studies, two endpoints, including “invisible spot on ophthalmoscopy” and “visible spot on fluorescein angiography”, have been used as the standard titration method for producing adequate SRT spots, given the differences in laser energy absorption due to individual variation in retinal pigmentation [24]. Several specific titration devices, such as optoacoustic dosimetry and reflectometry, have been developed to replace FFA [25,26]; however, their accuracy does not reach that of the standard titration method.

In a previous study using the standard titration method, pulse energy was varied using a fixed number (*n* = 30) of micropulses [24]. Various real-time dosimetry techniques have been developed in such a way that, out of 15 micropulses of ramping energy, 1 micropulse that finally reaches the threshold of RPE damage is considered therapeutically effective [26,27]. In addition to varying the pulse energy, the number of micropulses was controlled according to the findings on fundus images. We hypothesized that this approach would be useful in SRT for CSC. The present study aimed to evaluate the clinical outcomes of SRT using fundus image-based titration in patients with CSC.

## 2. Materials and Methods

### 2.1. Study Design and Participants

We reviewed the medical records of 54 patients who underwent SRT for the treatment of CSC between June 2023 and November 2023 at our institution. This retrospective cohort study followed the guidelines outlined in the Declaration of Helsinki, and data were collected in accordance with and approved by the institutional review board of Yeouido St. Mary’s Hospital (number: SC24RISI0047) of the Catholic University of Korea. All patients provided written informed consent after receiving an explanation of the potential risks associated with SRT, including retinal hemorrhage, retinal burns, and vision loss.

We included SRT-treated patients who (1) had eyes with the presence of SRF in the fovea for ≥3 months on optical coherence tomography (OCT); (2) showed focal or diffuse hyperfluorescent leakages on FFA or ICGA; (3) were aged 20–65 years; (4) had ≥6 months of medical records following SRT available. We excluded eyes with (1) the presence of other macular diseases, such as age-related macular degeneration and polypoidal choroidal vasculopathy; (2) a history of conventional laser or PDT; (3) the presence of retinal exudates and hemorrhage, indicating CNV; (4) a history of intravitreal anti-VEGF injection within 12 weeks pre-SRT; and (5) a history of local or systemic steroid use within 1 year pre-SRT.

### 2.2. SRT Procedure

**Figure 1:**
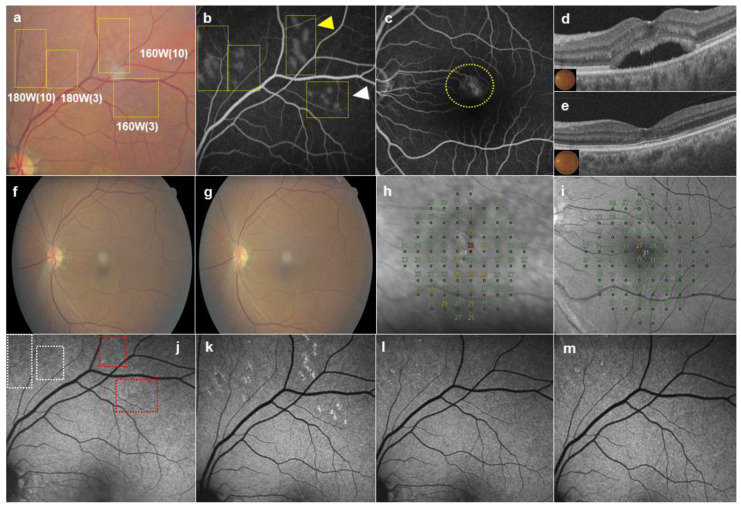
A 58-year-old female patient presented with a 12-month history of subretinal fluid (SRF) in the left eye. (**a**) By 1 h after irradiation, 20 test spots (yellow boxes) were barely visible on color fundus photography (CFP). Test spots made with three micropulses at 160 W of pulse energy were less barely visible than those produced with 10 micropulses at 160 W. (**b**) All barely visible test spots showed leakages on fundus fluorescein angiography (FFA). Test spots produced with three micropulses at 160 W of pulse energy (white arrowhead) showed less leakage than those made with 10 micropulses at 160 W (yellow arrowhead). (**c**) Thirty-five selective retina therapy (SRT) spots, at one-spot spacing, were produced by 3 micropulses (160 W) in the leakage area (yellow circle) on FFA. (**d**) SRF was observed on optical coherence tomography (OCT) at baseline. (**e**) SRF was resolved completely at 6 months post-SRT. No SRT spots were visible on CFP at baseline (**f**) and at 6 months post-SRT (**g**). (**h**) The mean deviation (MD) of microperimetry improved from −1.42 dB at baseline to +0.15 dB (**i**) at 6 months. (**j**) Hypoautofluorescent changes at 1 h post-SRT were observed at the test spots produced by 180 W micropulses (white box), but no change was seen at spots produced by 160 W micropulses (red box). (**k**) Autofluorescence of test spots had increased by 1 month post-SRT, but was decreased at 3 months (**l**) and 6 months (**m**) post-SRT. No scotomatous change was observed in the SRT-treated area on microperimetry.

A single surgeon (YJR) performed SRT using an SRT device (Macufocus; Threshold, Seoul, Republic of Korea), Q-switched Nd:YLF 527 nm laser with a spot-size diameter of 200 μm, single micropulse duration of 1.7 μs, and a pulse-repetition rate of 100 Hz. The Macufocus has been approved by the Ministry of Food and Drug Safety in South Korea for the treatment of CSC and diabetic macular edema. A retinal lens (Ocular Mainster Focal/Grid; Ocular Instruments, Bellevue, WA, USA) was used to deliver a 200 µm diameter spot onto the retina. In addition to being able to adjust the power of the pulsed laser, the number of micropulses (*n* = 1–30) of the same power could be adjusted according to the laser settings. We applied 20–30 preliminary test spots with 20 W increments of pulse energy (100–240 W) around the arcade vessels to determine the appropriate energy for the treatment spots. A fixed number of micropulses, either 3 or 10, in pairs of the same pulse energy, was used to investigate the changes caused by the difference in the number of micropulses. One hour after test spot irradiation, FFA was performed to determine the pulse energy required for treatment, based on two endpoints: ophthalmoscopically invisible and FFA-visible lesions. While an SRT spot is ophthalmoscopically invisible during irradiation, “barely visible” spots were observed on color fundus photography (CFP) 1 h after irradiation. The lowest power that produced a barely visible spot among the test spots irradiated with a bundle of 10 micropulses was selected as the power used for creating the treatment spot. However, the number of micropulses was reduced to a bundle of three micropulses for all treatment spots. SRT was applied to the leakage region on FFA, with a one-spot-spacing density (Figure 1). Retreatment was performed 3 months after SRT using the same laser parameters as the first treatment if SRF remained in the central macular area on OCT. However, if SRF was almost resolved (SRF height < 10 μm), no retreatment was performed.

### 2.3. Clinical Measures

To evaluate the response to SRT, all patients underwent slit-lamp examination and determination of the Snellen best-corrected visual acuity (BCVA) at SRT initiation and at 1, 2, 3, 4, and 6 months after treatment. The Snellen BCVA converted to the logarithm of the minimum angle of resolution (logMAR) for analysis. After pupil dilatation, all patients underwent examinations using multimodal imaging studies, such as CFP (CF-60UVi; Canon Inc., Ota, Japan), FFA, ICGA, fundus autofluorescence (FAF) (HRA2; Heidelberg Engineering, Dosenheim, Germany), and swept-source OCT (DRI OCT Triton, Topcon, Tokyo, Japan). OCT was used to identify the presence of SRF and to measure both the central foveal thickness (CFT) and SRF height (the maximum distance between the outer neurosensory retina and the RPE at the center of the fovea), utilizing scanning with a 7 × 7-mm^2^ volume centered on the fovea. Retinal layers were confirmed using IMAGENET 6.0 software (Topcon). The degrees of SRF resolution after SRT were categorized into three groups based on the rate of resolution of SRF height: complete resolution, ≥50% reduction, and <50% reduction in SRF height were classified as “complete response”, “partial response”, and “no response”, respectively.

CFP, FAF, and OCT images were obtained at baseline and at 1, 2, 3, 4, and 6 months after SRT. FFA and ICGA were performed to confirm the leakage points and diagnose CSC, respectively. The leakage type was classified as focal (1–3 leakage points) or diffuse (≥4 leakage points). On the treatment day, FFA was conducted 1 h after test spot irradiation to determine the preset pulse energy and the number of micropulses needed to produce the treatment spots. FFA was then repeated to confirm the leakage area for retreatment.

Retinal sensitivity was measured using microperimetry (compass fundus perimeter, CMP; Centervue, Padova, Italy), which consisted of a scanning ophthalmoscope, an infrared, red-free image of the posterior pole, and an automated perimeter with active retinal tracking. The mean deviation (MD), pattern standard deviation, pupil size, and false-positive rate were automatically calculated using compass fundus perimeter (CMP). The CMP test was regarded as reliable if the false-positive rate was ≤18% [28]. The CMP test was conducted at baseline and at 3 and 6 months after SRT.

### 2.4. Statistical Analysis

Changes in mean BCVA, CFT, and SRF height from baseline to 1, 2, 3, 4, and 6 months were analyzed using a paired *t*-test. The change in the mean MD of microperimetry from baseline to 3 and 6 months was analyzed using a paired *t*-test. Mean values with standard deviations are provided for all measurements. Statistical significance was defined as *p* < 0.05. Statistical analyses were conducted using SPSS v. 24.0 software (SPSS Inc., Chicago, IL, USA).

## 3. Results

Among the 54 eyes (52 patients) that underwent SRT for CSC during the study period, 29 eyes (29 patients) met the inclusion criteria. Patients were excluded for the following reasons: <6 months’ follow-up (15 eyes), history of anti-VEGF intravitreal injection within 10 weeks pre-SRT (5 eyes), history of steroid therapy within 1 year before SRT (2 eyes), history of conventional laser therapy (2 eyes), and history of PDT (1 eye). The mean age of patients was 50.5 ± 9.8 years (33–63 years). The mean number of CSC episodes among the patients was 3.64 ± 3.38, and the mean duration of the current episode was 34.21 ± 38.91 months. The percentage of patients who had undergone previous intravitreal bevacizumab injections was 65.5% (Table 1).

The mean BCVA (logMAR) improved significantly from baseline to 6 months post-treatment (*p* = 0.009; Table 2). The mean CFT and mean SRF height decreased significantly over this period (both *p* < 0.001; Table 2). While the mean SRF height increased slightly from 2 months to 3 months, it continuously decreased after retreatment at month 3. Retreatment was performed in 34.5% (10/29 eyes) of eyes at 3 months after initial SRT. The mean MD, calculated using CMP, improved from baseline to 6 months post-treatment, but the change was not statistically significant (*p* = 0.07; Table 2).

**Figure 2:**
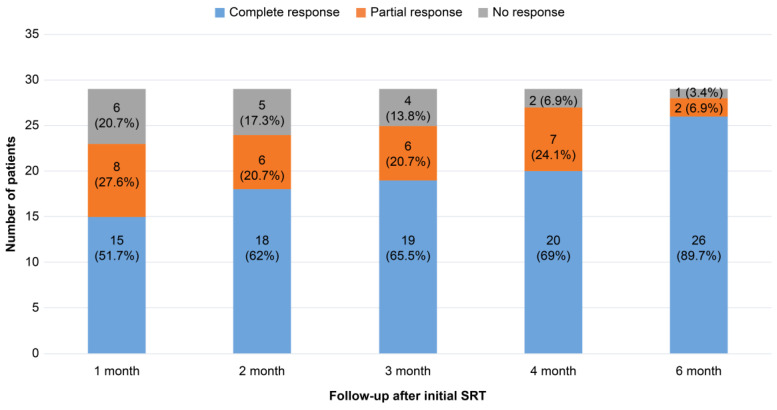
Subretinal fluid (SRF) resolution rate following selective retinal therapy (SRT) over a 6-month follow-up period. A decrease in SRF height of 100%, ≥50%, and <50% was categorized as complete, partial, and no response, respectively.

At 6 months post-SRT, 26 eyes (89.7%) showed complete SRF resolution, 2 eyes (6.9%) showed a partial response, and 1 eye (3.4%) showed no response (Figure 2).

In the initial SRT, the mean pulse power for treatment spots was 183.1 ± 24.07 W (range: 110–240 W). Although the mean pulse power of the treatment spots was the same as that of the barely visible test spots produced with 10 micropulses, all treatment spots were irradiated with 3 micropulses. The mean number of treatment spots for the initial SRT was 21.41 ± 13.84. All treatment spots were invisible during irradiation, and all barely visible SRT spots were not observed on CFP at 3 months post-SRT. Similar to a previous report, autofluorescence in the SRT-treated area showed no change or hypofluorescence by 1 h after irradiation, which changed to hyperautofluorescence after 1 month. While the autofluorescence of SRT spots gradually decreased over the 6-month follow-up period (Figure 1) in this study, some spots maintained hyperfluorescence surrounding a tiny central dark dot in a previous report [29]. Other laser-related adverse effects, such as retinal burns and hemorrhage, were not observed.

## 4. Discussion

In this study, we evaluated the clinical outcomes of SRT using a fundus image-based titration approach for treating CSC. Six months post-SRT, SRF was completely resolved in 89.7% of cases, and the mean BCVA had improved significantly from baseline. The mean CFT decreased significantly, and the mean MD improved from baseline to 6 months post-treatment.

In previous studies, SRT delivered using a standard treatment protocol or real-time feedback dosimetry showed a complete SRF resolution rate of 64.7–74% at 3 months post-SRT in CSC patients [25,26,27,30]. Although our study had different patient inclusion criteria and treatment methods compared to those used in previous studies, the complete SRF resolution rate at 3 months was 65.5%, which was similar to that of previous studies. Moreover, after repeated SRT treatment, the resolution rate improved to 89.7% at 6 months post-SRT. Therefore, our results demonstrated that SRT using fundus image-based titration is effective in removing SRF without SRT-related adverse events.

The two endpoints of SRT, invisible on ophthalmoscopy and visible on FFA, have been the standard guideline for appropriate SRT treatment. However, in our previous studies on Asian patients with CSC or with diabetic macular edema, although SRT spots were invisible for a few minutes during irradiation, “barely visible spots” were observed on CFP by 1 h after SRT. The barely visible spots were regarded as appropriate SRT spots because they became invisible on CFP over a 3-month period and did not show anatomical changes on OCT. Moreover, barely visible spots did not induce scotomatous changes on microperimetry [30,31]. In a previous report, no changes in FAF were observed immediately after SRT, due to minimal heat conduction in the neurosensory retina. However, autofluorescence in the SRT spot gradually decreased by 10 min after SRT, due to RPE degradation. One hour after SRT, the hypofluorescent spots were more pronounced, indicating RPE edema rather than photoreceptor damage. These hyperautofluorescence changes appear to be associated with higher metabolic uptake during RPE proliferation [29]. We deduced that the delayed “barely visible’’ changes caused by RPE swelling at 1 h after irradiation could be more noticeable in Asian patients because Asians have more fundus pigmentation, creating a higher contrast effect than in Caucasians, who have less fundus pigmentation. Thus, SRT using fundus image-based titration may be more useful for Asian patients than for Caucasian patients. However, the association between SRT spot visibility and racial differences in fundus pigmentation needs to be investigated in further studies.

In a previous study, the selectivity of photocoagulation was analyzed using a rate process model for thermal damage. Both the micropulse duration (or width) and repetition frequency are considered important parameters in selective retinal photocoagulation. Compared to a single micropulse duration of 50 μs or 100 μs, a micropulse duration of 2 μs showed no heat conduction. Higher repetition frequencies of 500 or 1000 Hz showed a temperature increase of up to 65 °C, while frequencies of 100 Hz showed a lower temperature increase of up to 40 °C [32]. Therefore, a 2 μs micropulse duration with a frequency of 100 Hz was regarded as an appropriate parameter for producing selective RPE damage.

Interestingly, regardless of the repetition frequency, most of the temperature increase occurred during the first 10 micropulses. Since a 2 μs micropulse duration showed a very small temperature increase within the first 10 micropulses, reducing the number of micropulses may be useful in producing a reduction in tissue reaction at threshold levels, damaging the RPE. Although a lower number of micropulses may produce less tissue reaction to SRT, the number of micropulses used for the titration of treatment spots was set to three in this study. In a previous rabbit-based experiment, a 1.7 μs duration for one micropulse showed a lower safety margin than a 1.7 μs with 10 micropulses. The safety margin factor is defined as the ratio of threshold energies producing 84% visible spots on FFA and 16% visible spots on ophthalmoscopy [33]. In our study, three micropulses, rather than one, were selected to obtain a wider safety margin. Although other titrations with different numbers of micropulses were possible, the reduction in RPE damage with three micropulses was clearly demonstrated for CFP and FFA in the present study.

**Figure 3:**
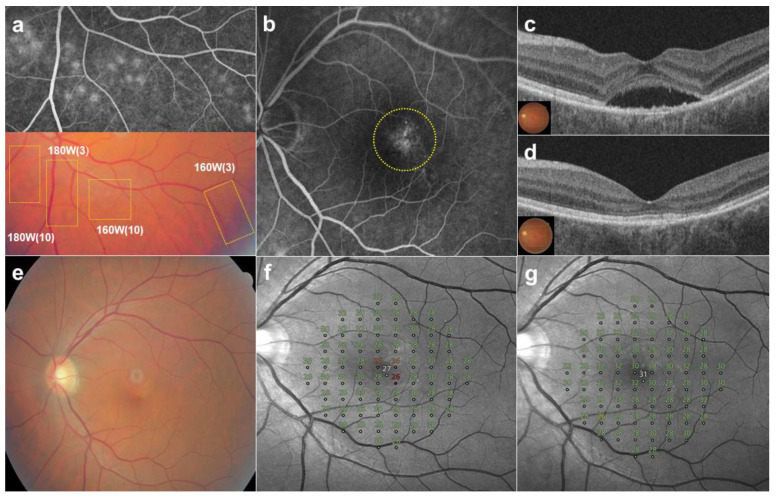
A 52-year-old male patient presented with a 9-month history of subretinal fluid (SRF) in the left eye. (**a**) Twenty test spots (yellow boxes) were observed at 1 h after irradiation on color fundus photography (CFP). While test spots (160 and 180 W) delivered with 10 micropulses were ”barely visible”, other test spots produced with 3 micropulses were invisible on CFP. All test spots showed leakages on fundus fluorescein angiography (FFA). (**b**) Thirty-nine SRT spots (160 W) with one-spot spacing, produced with three micropulses, were applied at diffuse leakages (yellow circle) on FFA. (**c**) SRF was seen on optical coherence tomography (OCT) at baseline (**d**). SRF was resolved completely on OCT at 6 months post-SRT. (**e**) No SRT spots were visible on CFP at 6 months post-SRT. (**f**) The mean deviation (MD) of microperimetry improved from −0.45 dB at baseline to −0.10 dB (**g**) at 6 months after SRT. No scotomatous change was observed in the SRT-treated area on microperimetry.

In the endpoint management protocol using a subthreshold laser, a barely visible lesion was defined as 100% laser power. Endpoint management is performed at 30% energy, which is a therapeutic, non-damaging setting for macular diseases [34]. As a different form of endpoint management, the therapeutic setting of the fundus image-based SRT in this study was a 70% reduction in the number of micropulses. Although barely visible spots were used as the criterion for fundus image-based SRT, FFA is still useful for identifying target leaks in the macular area and for assessing the extent of leakage, based on the difference in micropulse counts between the test spots. While the barely visible lesion from a bundle of 10 micropulses had 100% laser power, the number of micropulses was set to 3 (30%) for macular treatment in this study. At the same power, SRT spots from 3 micropulses were less visible (Figure 1) or invisible (Figure 3) on CFP and showed less leakage on FFA, as compared to those obtained with 10 micropulses. Although fundus image-based SRT also uses the two standard endpoints (invisible on ophthalmoscopy and visible on FFA), in addition to the control of pulse power, the control of micropulse counts was useful for titration. Because less leakage of FFA in the SRT spot indicates less RPE damage, fewer barely visible and invisible spots produced with 3 micropulses can be more favorable for SRT treatment than barely visible spots produced with 10 micropulses.

**Figure 4:**
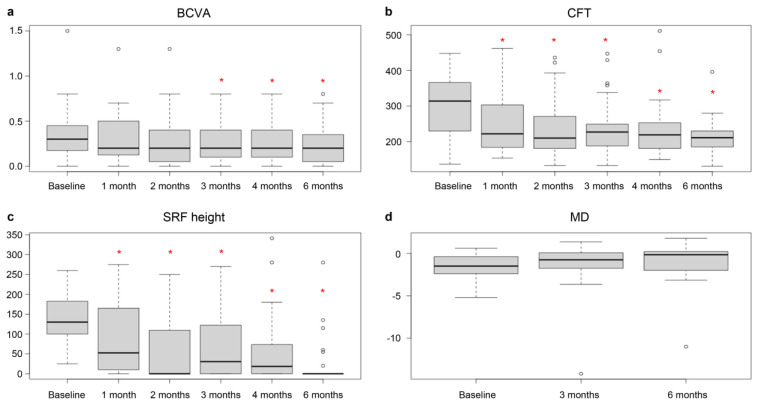
Box plots of changes in (**a**) best-corrected visual acuity (BCVA), (**b**) central foveal thickness (CFT), (**c**) subretinal fluid (SRF) height, and (**d**) mean deviation (MD) during the 6-month follow-up period after selective retinal therapy (SRT). * *p* < 0.05.

In our study, SRT using fundus image-based titration was not only effective in removing SRF but also in improving BCVA. After SRT, the mean CFT and mean SRF height significantly reduced at 1 month, whereas the mean BCVA significantly increased at 3 months, suggesting that functional improvement occurred later than anatomical improvement. As the complete SRF resolution rate and mean SRF height improved further after retreatment at 3 months, retreatment may be helpful even when the initial treatment shows no response (Figure 4). Additionally, CMP showed an increase in MD, indicating the photoreceptor-sparing effect of SRT.

Our study had several limitations, including its retrospective design, small number of patients, short follow-up period, and bias due to single-surgeon procedures. Additionally, we did not include data on choroidal thickness changes because no significant changes in choroidal thickness were found after SRT in our previous studies [26,27].

## 5. Conclusions

Our results support the concept that SRT using fundus image-based titration may be effective for treating CSC patients. However, further studies with larger and more diverse populations are needed to confirm the effectiveness of SRT using fundus image-based titration.

## Figures and Tables

**Table 1:** Baseline demographics and characteristics of 29 eyes in 29 patients with central serous chorioretinopathy.

Patients’ Characteristics	Values
Patients (eyes), *n*	29 (29)
Age (years), mean ± SD	50.5 ± 9.8
Sex, *n* (%)	Male 24 (82.8%), Female 5 (17.2%)
Duration of current episode (months), mean ± SD	34.21 ± 38.91
Number of episodes (*n*), mean ± SD	3.64 ± 3.38
Number of patients who underwent previous intravitreal bavacizumab injection, *n* (%)	19 (65.5%)
BCVA (logMAR), mean ± SD	0.34 ± 0.31
CFT (μm), mean ± SD	309.31 ± 81.6
SRF height (μm), mean ± SD	138.36 ± 56.78
MD (dB) of microperimetry, mean ± SD	−1.56 ± 1.47

BCVA, best-corrected visual acuity; CFT, central foveal thickness; SRF, subretinal fluid; MD, mean deviation; SD, standard deviation.

**Table 2:** Changes in BCVA, CFT, SRF height, and MD during the 6-month follow-up period after SRT.

	Baseline	1 Month	2 Months	3 Months	4 Months	6 Months
**BCVA (logMAR)**						
Mean	0.34	0.33	0.31	0.26	0.25	0.24
SD	0.31	0.3	0.31	0.24	0.21	0.24
*p* value		0.499	0.177	0.026 *	0.007 *	0.009 *
**CFT (μm)**						
Mean	309.31	257.79	244.62	239.41	234.38	211.07
SD	81.6	96.8	86.44	78.11	81.55	50.21
*p* value		0.003 *	<0.001 *	<0.001 *	<0.001 *	<0.001 *
**SRF height (μm)**						
Mean	138.36	85.93	63.5	64.11	51.68	23.75
SD	56.78	88.22	85.18	79.57	85.26	61.19
*p* value		0.002 *	<0.001 *	<0.001 *	<0.001 *	<0.001 *
**MD (dB)**						
Mean	−1.56			−1.20		−1.03
SD	1.47			2.86		2.43
*p* value				0.374		0.07

BCVA, best-corrected visual acuity; CFT, central foveal thickness; SRF, subretinal fluid; MD, mean deviation; SRT, selective retinal therapy; SD, standard deviation. * *p* < 0.05.

## Data Availability

The data presented in this study are available on request from the corresponding author.
